# Monogenic Causes of Strokes

**DOI:** 10.3390/genes12121855

**Published:** 2021-11-23

**Authors:** Justyna Chojdak-Łukasiewicz, Edyta Dziadkowiak, Sławomir Budrewicz

**Affiliations:** Department of Neurology, Wroclaw Medical University, 50-556 Wroclaw, Poland; edyta.dziadkowiak@umw.edu.pl (E.D.); slawomir.budrewicz@umw.edu.pl (S.B.)

**Keywords:** stroke, ischemic stroke, hemorrhagic stroke, genetics

## Abstract

Strokes are the main cause of death and long-term disability worldwide. A stroke is a heterogeneous multi-factorial condition, caused by a combination of environmental and genetic factors. Monogenic disorders account for about 1% to 5% of all stroke cases. The most common single-gene diseases connected with strokes are cerebral autosomal dominant arteriopathy with subcortical infarcts and leukoencephalopathy (CADASIL) Fabry disease, mitochondrial myopathy, encephalopathy, lactacidosis, and stroke (MELAS) and a lot of single-gene diseases associated particularly with cerebral small-vessel disease, such as *COL4A1* syndrome, cerebral autosomal recessive arteriopathy with subcortical infarcts and leukoencephalopathy (CARASIL), and Hereditary endotheliopathy with retinopathy, nephropathy, and stroke (HERNS). In this article the clinical phenotype for the most important single-gene disorders associated with strokes are presented. The monogenic causes of a stroke are rare, but early diagnosis is important in order to provide appropriate therapy when available.

## 1. Introduction

Strokes are the most common cause of death and disability worldwide. Globally, the incidence of strokes has substantially increased in recent decades, as a result of ageing populations and the increased prevalence of many modifiable stroke risk factors [1]. The pathogenesis of a stroke is multi-factorial and incompletely understood. According to the TOAST (trial of ORG 10172 in acute stroke treatment) classification, the etiology of a stroke is divided in five categories: large vessel disease (extracranial and intracranial), small vessel disease, cardiovascular embolism, strokes of other etiology and strokes of unspecified etiology [2]. Genetic factors are more responsible for small- and large-vessel disease than in the cardio-embolic etiology of a stroke. In every category we can distinguish both the sporadic and genetic form. Many different types of disease like strokes can be passed down through families. In some cases, a stroke is a part of a systemic disorder, while others are limited only to the nervous system [3]. The monogenic condition is responsible for 1% of strokes, especially in younger patients, but the data is underestimated [4]. The real genetic impact of the origin of strokes is still unknown.

## 2. Methods

The aim of the paper was to find the relationship between strokes and monogenetic disorders. The key search terms applied in PubMed via MEDLINE and Google Scholar were “stroke” and “ischemic” or “hemorrhagic” and “genetic” or “monogenic”. The reference lists from eligible publications were searched online for their relevance to the topic. We considered publication records until July 2021. Reviews and research studies, classified according to their relevance, were included.

## 3. Results

Table 1 shows the most common monogenic causes of ischemic and hemorrhagic strokes. The arteriopathies (CADASIL and CARASIL), metabolic diseases (Fabry, homocystinuria) and connective tissue disorders (Ehlers-Danlos syndrome type IV) can involve an ischemic stroke as part of the phenotype. The genetic form of cerebral amyloid angiopathy or mutations of soft tissue can be connected with the increased risk of intracranial bleeding.

### 3.1. CADASIL

CADASIL (cerebral autosomal-dominant arteriopathy with sub-cortical infarcts and leukoencephalopathy) is an autosomalously inherited micro-angiopathy with onset in adulthood. It is characterized by arteriopathy, sub-cortical infarcts and leukoencephalopathy. Common clinical manifestations are recurrent strokes, migraine or migraine-like headaches, progressive dementia, pseudo-bulbar paralysis and psychiatric conditions [5]. CADASIL is caused by a missense mutation in the *NOTCH3* gene on chromosome 19p13. The *NOTCH3* gene consists of 33 exons. The pathogenic mutations of the *NOTCH3* gene in CADASIL are located in exons 2–24 coding for the 34 EGFr (epidermal growth factor-like repeat) domains. Wang et al. identified a novel mutation, R1761H (c.5282G>A), on exon 29 of the *NOTCH3* gene in a Chinese family [6]. Encoded by the *NOTCH3* gene, the protein is a large trans-membrane receptor involved in intercellular communication. It is found in vascular smooth muscle cells. Some reports indicate that pericytes—the other major form of *NOTCH3*-expressing mural cell in vasculature—are also affected. Notch3 signaling may also play a role for the astrocyte number [7,8]. Typical of CADASIL on MR imaging, white matter hyper-intensity (WMH) in CADASIL is localized in the white matter of the anterior temporal poles, external capsules, and superior frontal regions. De Guio et al. have shown different types of white matter hyperintensity in CADASIL, based on a 7-Tesla MRI examination, using high-resolution images and analyses of relaxation times (T1R: longitudinal relaxation time and T2*R: effective transversal relaxation time). The authors concluded that the specific white matter hyperintensities differ from those observed in other non-specific white matter areas. The results suggest large differences in water content between specific and non-specific WMH in CADASIL, supporting the idea that mechanisms underlying WMH may differ according to their location [9].

The deposition of granular osmiophilic material (GOM) in the vascular wall is considered a hallmark of the disease [10].

### 3.2. CARASIL

CARASIL (cerebral autosomal recessive arteriopathy with sub-cortical infarcts and leukoencephalopathy) is an inherited systemic disorder in which there is damage to the small blood vessels in the brain, brain stem and hair follicles. The main clinical manifestations of CARASIL are ischemic stroke or stepwise deterioration in brain functions, progressive dementia, premature baldness, and attacks of severe low back pain or spondylosis deformans / disk herniation. CARASIL (Maeda syndrome) was introduced by Maeda as a very rare disease in Japan. Other synonyms for the name CARASIL are Nemato disease and familial young-adult-onset arteriosclerotic leukoencephalopathy with alopecia and lumbago without arterial hypertension [11,12].

CARASIL is a single-gene disorder directly affecting the cerebral small blood vessels that is caused by mutations in the *HTRA1* gene encoding HtrA serine peptidase / protease 1 (HTRA1). HTRA1 disorder caused by biallelic pathogenic variants (i.e., the classic CARASIL phenotype) is inherited in an autosomal recessive manner. HTRA1 disorder caused by heterozygous pathogenic variants is inherited in an autosomal-dominant manner. HTRA1 disorder includes two phenotypes—classic CARASIL and HTRA1 cerebral small vessel disease (HTRA1-CSVD) [13]. High temperature requirement protein A1 (HtrA1) is a primarily secreted serine protease involved in a variety of cellular processes, including transforming growth factor β (TGF-β) signaling. Impaired TGF-β signaling, due to a lack of LTBP-1 processing by HtrA1, is thought to promote the pathogenesis of CARASIL, but the underlying molecular mechanisms are not fully understood [14]. The most characteristic findings on brain magnetic resonance imaging are diffuse white matter changes and multiple lacunar infarctions in the basal ganglia and thalamus [11]. The MRI scan can reveal diffuse leukoencephalopathy with multiple lacunar infarctions. In T2-weighted sequences, diffuse, symmetric lesions with hyperintense signals can be seen in the white matter, typically in the periventricular white matter and deep white matter. In some patients, there is anterior involvement of the temporal lobes and external capsule, which are confluent since the initial stage of the disease. T2*-weighted sequences also showed areas with hemosiderin deposits. A typical sign of this disease in the later stages is the “split pons sign”, consisting of hyperintense lesions in the pons [15]. Histopathologically, CARASIL is characterized by intense arteriosclerosis, mainly in small penetrating arteries, without granular osmiophilic materials or amyloid deposition [11]. Histopathological studies have also described changes in skin arteries that included intimal proliferation or loss of smooth muscle cells in small arteries [16].

### 3.3. RVCL-S

Retinal vasculopathy with cerebral leukoencephalopathy and systemic manifestations (RVCL-S) is a small-vessel disease that affects highly vascularized tissues including the retina, brain, kidneys, and liver. This syndrome is also referred to as cerebroretinal vasculopathy (CRV), hereditary systemic angiopathy (HSA), hereditary endotheliopathy, retinopathy, nephropathy and stroke (HERNS), hereditary vascular retinopathy (HVR), TREX1 vasculopathy, retinal vasculopathy with cerebral leukodystrophy (RVCL). This is a rare genetic disease with an autosomal-dominant inheritance pattern that causes progressive loss of tiny blood vessels, ultimately resulting in visual deterioration and a series of mini-strokes in the brain [17,18].

The gene responsible for causing RVCL-S, called TREX1, encodes a 3′-exonuclease 1 protein that removes nucleotides from the 3′ ends of DNA molecules to remove unneeded fragments that may form during DNA replication. A mutation C-terminal frameshift in the tail end of TREX1 produces a shortened version of the protein that mislocalizes inside cells. Muted TREX1 protein maintains DNase activity, but aberrant localization of muted protein due to impaired translocation into the nucleus in response to oxidative DNA damage may be associated with systemic micro-vascular endotheliopathy in patients with RVCL. Malfunctioning of TREX1 is associated with a broad spectrum of inflammatory and autoimmune diseases which are apparently independent, such as Aicardi-Goutieres syndrome (AGS), cryofibrinogenemia, familial chilblain lupus (FCL), systemic lupus erythematosus (SLE) and retinal vasculopathy with cerebral leukodystrophy (RVCL) [17,19,20,21,22].

Thus, due to the rare occurrence of RVCL-S, MR imaging studies were limited to small case series and showed small/medium punctate T2 hyperintense white matter lesions, some associated with diffusion restriction or nodular enhancement, and annular (rim- enhancing) pseudotumor lesions, often seen in advanced disease and causing life-threatening mass effect due to angioedema. Yan et al. presented a case report of a patient with RVCL-S who underwent follow-up magnetic resonance imaging at two-year follow-up, revealing a pattern of progression of brain lesions [23]. Sequential MR neuroimages showed small punctuate lesions with or without nodular enhancement and pseudotumor lesions enhancing the periphery. The lesions were located in both supratentorial and infratentorial white matter areas. The periventricular lesions showed a much larger size than the cerebellar lesions. Central diffusion restriction, peripheral rim enhancement and microbleeding, and massive swelling around the lesion, persisting for several months or even longer, were noted in these lesions with an aggressive appearance. Brain MRI findings provide some insight into the pathomechanism of RVCL-S: diffusion-limiting punctate lesions argue for a role of microvascular ischemia, whereas nodular and ring-enhancing lesions suggest a disruption of the blood-brain barrier. Cerebral pathology in RVCL-S shows microvascularity associated with a multi-laminated basement membranes, luminal stenosis, mural thickening and hyalinisation, and fibrinoid necrosis, which can resemble radiation necrosis. Chronic inflammatory cells may be seen close to the microvasculature or rarely in the parenchyma. In post-mortem brain tissue from patients with RVCL-S, TREX1 is readily detectable within the microglia surrounding the white matter microvasculature, suggesting that mislocalization of TREX1 may play a role in vulnerability to white matter damage [22,24,25].

### 3.4. MELAS

MELAS (mitochondrial encephalomyopathy with lactic acidosis and stroke-like episodes) belongs to the area of mitochondrial diseases, determined by mutations in mitochondrial DNA and inherited in the maternal line. There are two groups of mutations in mtDNA—one type of mutation affects general mitochondrial protein biosynthesis (tRNA and rRNA genes), exemplified by the 3243A→G nucleotide mutation in the mitochondrial tRNALeu gene; the other type of mutation causes amino acid substitutions in enzyme complexes involved in OXPHOS (protein coding genes). Dysfunction of respiratory complex I (NADH dehydrogenase type I: ND), which plays an important role in OXPHOS complexes, is considered one of the main mechanisms underlying mitochondrial diseases, which include MELAS [26,27,28]. The literature highlights the genetic heterogeneity of MELAS syndrome [29]. The involvement of nuclear gene mutations in MELAS syndrome was also demonstrated. Phenotype analysis and hierarchy of causative mutations identified deletion mutations encoding proteins in nuclear genes with a known mitochondrial function, previously described in mitochondrial disorders (POLG, DGUOK, SUCLG2, TRNT1) [30,31,32]. A study by Chakrabarty et al. found both nuclear (POLG) and mitochondrial (MT-TL1, m.3243A>G) mutations in two patients with MELAS, confirming the role of dual genetic mutations in the MELAS phenotype [30]. The authors believe that the contribution of a nuclear genetic background may influence the clinical heterogeneity in m.3243A > G-related classical mitochondrial disease.

In 80% of patients, the disease is caused by an A3243G point mutation involving the conversion of adenine to guanine at position 3243 within the *MTTL1* gene (OMIM # 590050), which encodes a tRNA for leucine. Other genes whose mutations lead to MELAS include MTTQ, MTTH, MTTK, MTTS1, MTND1, MTND5, MTND6 and MTTS2. Case reports with different mutations are presented in the literature, e.g., a base and amino acid exchange mutation in the *MT-ND4* gene (m.12015T>C on p.Leu419Pro) and the 14453G→A mutation with associated mutations in the D-loop regions of mitochondrial DNA [26,33].

MELAS is associated with a failure to thrive, lactic acidosis, neuromyopathy, epilepsy, migraine-like headaches and recurrent stroke-like episodes (SLEs) resembling vaso-occlusive strokes [34]. Fluctuating, migratory stroke-like lesions with a predilection for the parietal, temporal, and occipital cortex that do not conform to a vascular territory and a lactate spike at 1.3 ppm on MR spectroscopy are characteristic of MELAS syndrome [35]. SLEs develop subacutely over hours to days (or weeks), have a high potential for reversibility and complex hemodynamic changes. The current literature points to neuronal and/or glial damage due to mitochondrial failure and cerebrovascular angiopathy with dysregulated cerebral perfusion [34]. The stroke-like episodes are related to vasogenic edema, hyperperfusion and neuronal damage. Acute oxidative phosphorylation defect may have a crucial role in the pathophysiology [36,37,38]. In the study by Ito et al., extensive hemorrhagic petechiae along the veins of the cerebral cortex corresponding to acute stroke-like lesions were described in the acute phase of SLEs [36]. In the chronic phase of stroke-like episodes, foci of necrosis and specific vascular changes (mitochondrial abnormalities in small arteries) were found.

MR imaging has also been shown to have features characteristic of the acute and chronic phases associated with stroke-like episodes. In the acute phase, gyral swelling, gyriform cortical diffusion restriction, subcortical white matter T2 FLAIR hyperintensity with elevated ADC values and elevated parenchymal lactate levels on MR spectroscopy were described in acutely affected and non-affected brain regions. The anatomical distribution of acute lesions is typically in the occipital, parietal and posterior temporal lobes and cerebellar hemispheres, although involvement of the lateral temporal cortex, mesial temporal lobes and posterior frontal cortex has also been described, without concordance with a single vascular territory [39,40,41]. Classic involvement of the pericalcarine visual cortex in the medial occipital lobes is associated with episodes of hemianopia or cortical blindness. Cortical involvement was usually described as asymmetrical; however, cases with symmetrical involvement are increasingly being recognized [42,43]. Lesions frequently spread to the cortex of adjacent gyri over time in a migratory fashion, resulting in large regions of cortical involvement that cross the boundaries of arterial vascular territories, often associated with seizures. These diffuse signal abnormalities are likely to resolve over time, in parallel with clinical improvement, while new lesions may develop with or without new symptoms. This wandering, waxing and waning pattern of stroke-like lesions on imaging studies is a fundamental and characteristic feature of MELAS syndrome [35,44]. On angiography, the blood vessels are usually normal, but vasoconstrictions have occasionally been described [45]. In the subacute phase, cortical lesions may be characterized by T2 hypointensity (“black toenail sign”) and T1 hyperintensity in gyral areas, indicating cortical laminar necrosis. The laminar nature of the necrosis preferentially spares the subpial superficial cortical layers, with the greatest involvement of the neuron-rich middle cortical layers (layers III–V) [46,47]. In the chronic phase, venous infarcts develop into areas of encephalomalacia, volume loss and progressive multifocal cerebral and cerebellar atrophy, with associated cognitive decline. Symmetrical calcifications in the basal ganglia have also been described [48].

### 3.5. Sickle Cell Disease (SCD)

Sickle cell disease is the most common inherited red blood cell disorder, caused by point mutation (GAG to GTG) in the ß-globin gene, resulting in the structural abnormality of haemoglobin (called haemoglobin S-HbS) [49]. SCD is much more prevalent in African-Americans than people of other ethnicities. The disease is widespread in sub-Saharan Africa, in the Middle East, the Indian subcontinent, and some Mediterranean regions [50]. The course of sickle cell disease is heavy and burdensome, due to a variety of acute and chronic complications. Clinical manifestations of SCD are mainly characterized by chronic haemolysis and acute vaso-occlusive crisis, which are responsible for acute and chronic organ damage. SCD is connected with a higher risk of stroke. The estimated prevalence of stroke is about 3.75%, especially in the first decade of life, due to a high flow rate and vulnerability of changes of endothelium in this age group [51]. The abnormal red cells force the endothelial shear pressure of the vessel, damage the surface and cause occlusion of the large vessel leading to ischemic stroke [52,53]. Ischemic strokes are often located in border zone regions [54]. Some studies have shown a high prevalence of the development of aneurysm, they are often multiple and localized in the posterior circulation. Patients with SCD and aneurysm are at increased risk of ruptures, even in cases of smaller sizes [55].

### 3.6. Ehlers-Danlos Syndrome Type IV (EDS-IV)

Ehlers-Danlos syndrome type IV, also known as vascular Ehlers-Danlos syndrome, is an inherited condition associated with abnormal procollagen III synthesis resulting from heterozygote mutations in the *COL3A1* gene [56]. The abnormal type III collagen has reduced strength, elasticity and healing properties [57]. The estimated prevalence of EDS type IV is about 1 in 100,000 to 1 in 250,000 in the general population, without preference to sex or ethnicity [58]. EDS-IV is characterized by a facial dysmorphy (acrogeria), skin symptoms (thin and translucent skin with highly visible subcutaneous vessels), ecchymoses and haematomas, and arterial, digestive and obstetrical complications. Vascular complications are common and affect all anatomic areas, with preference to medium- and large vessels. Cerebrovascular events include intracranial aneurysms (typically in the cavernous sinus), dissection of vertebral and carotid arteries in intra- and extracranial segments and spontaneous rupture of large and medium-sized arteries [59,60]. Arterial abnormalities such as tortuosity, ectasia and dilatation or stenosis have been observed in EDS Type IV.

### 3.7. Homocystinuria

Homocystinuria is an autosomal recessive disorder that affects the metabolism of the amino acid methionine. Prevalence has been estimated to be 1 in 344,000 [61]. Type I is associated with a defect in the transsulfuration pathway or methylation pathway (types II and III). The defect in the methionine metabolism, leads to an abnormal accumulation of homocysteine and its metabolites (methionine, homocysteine, and their S-adenosyl derivatives) in the blood and urine. Homocystinuria type I is caused by the deficiency of the enzyme cystathionine-β-synthase (CBS), which converts homocysteine to cystathionine in the transsulfuration pathway of the methionine cycle and requires pyridoxal 5-phosphate as a cofactor [62]. The clinical manifestation is heterogenous, and includes eye, skeleton, nervous system, and vascular system abnormalities. Vascular events are the most serious complications in patients. Approximately 40% of patients with homocystinuria have a thromboembolic vascular lesion. The mechanism is not clear, but probably thrombosis and atherosclerosis are secondary to endothelial injury and stimulation of platelet aggregation [63]. Premature cerebrovascular disorders are the most common cause of death and disability before the age of 20 [61].

### 3.8. Pseudoxantoma Elasticum

Pseudoxantoma elasticum, also known as Groenblad-Strandberg syndrome, is an autosomal recessive elastic tissue disease, caused by mutations in the *ABCC6* gene [64]. The lack of functional *ABCC6* protein leads to ectopic mineralization of soft connective tissue, resulting in fragmentation of elastic fibers, involving mainly the skin, eyes and cardiovascular system. The prevalence is estimated at somewhere between 1 in 100,000 and 1 in 250,000 with a predominance in females. Patients with PXE have are at increased risk of vascular events associated with accelerated arteriosclerosis. The media and intima of blood vessels (mainly small- and medium-sized arteries) are affected by the abnormal mineralization of connective tissue [65]. The prevalence of ischemic stroke in PXE is more common than in the general population. A stroke is caused by occlusion of large or small vessel disease. Abnormalities in the arterial walls are the cause of hypertension and thus predisposed to cerebrovascular events.

### 3.9. Fabry’s Disease

Fabry’s disease is a rare, genetically determined, X-linked, recessive lysosomal storage disease caused by mutation in the *GLA* gene encoding enzyme alpha-galactosidase A (α-Gal-A) [66,67]. A lack of or a deficiency of enzyme results in progressive accumulation of glycosphingolipids, especially globotriaosylceramide in the heart, renal epithelium, skin, eye, and vascular system. The incidence of the disease is estimated at about 1 in 40,000–60,000 male patients per year [67]. The clinical picture is very varied and characterized by high inter-individual variability [68]. The neurological manifestation of the disease includes peripherial polyneuropathy, autonomic dysfunction and cerebral manifestation. The incidence of cerebrovascular events is approximately 40%, especially strokes in the posterior circulation have been observed [69]. A stroke may be the first manifestation of the disease, as approximately 20% patients before a stroke suffered a TIA. Based on data from the Fabry Registry, the incidence of stroke was estimated much higher than the general population, in males at about 6.9%, and 4.3% in females [69]. In the case of homozygotes, cerebral symptoms appear earlier, at around 34 years (33.8), and in the case of heterozygotes at the age of 40 [67]. The mechanism of a stroke is unclear, studies having shown that most strokes are secondary to small vessel disease [67]. Another possibly vascular complication is dilative arteriopathy of the vertebrobasilar circulation [70].

### 3.10. Marfan Syndrome

Marfan syndrome is an autosomal dominant condition of connective tissue, associated in 90% of cases with mutations on the fibrillin-1 (*FBN1*) gene. The incidence of Marfan’s syndrome is estimated at about 2–3 per 10,000. The clinical characteristic of the disease includes cardiovascular, skeletal and ocular symptoms. The diagnosis is based on a consensus established at the International Nosology of Heritable Disorders of Connective Tissue Meeting in Berlin in 1986. Cardiovascular manifestations are conveniently put into two groups: dysfunction of the heart, and vascular problems [71]. Cardiovascular complications occur in most patients with Marfan syndrome, aneurysm and dissection of aorta are cardinal clinical features of the disease [72,73]. Neurovascular complications are rare, as only 10–20% of patients with Marfan syndrome are observed [74]. Several possible mechanisms in the etiology of strokes are observed. Abnormalities in fibrillin in medium-sized and large arteries lead to the disruption of elastic fibers and formation of aneurysm or dissection of the artery. Extension of proximal aortic dissection into the brachiocephalic or spinal arteries is one potential mechanism for the onset of a stroke. In some patients, embolism secondary to prosthetic heart valves and atrial fibrillation is connected with a stroke [75].

### 3.11. COL4A1 and COL4A2-Associated Syndromes

Dysfunctions of collagen type IV α1 and α2 are autosomal-dominant conditions, caused by mutations in the *COL4A1* (13q34) and *COL4A2* (13q34) genes. The spectrum of clinical features is very heterogenous and includes neurologic features (stroke, migraine, infantile hemiparesis, epilepsy) and systemic symptoms such as eyes, kidneys, heart and muscle abnormalities [76].

Autosomal-dominant early-onset cerebral small vessel disease is associated with the *COL4A1* gene mutations [77]. The disease manifests itself in the fourth decade of life with heterogenus clinical features. Diffuse leukoaraiosis, microbleeds, lacunar stroke, dilated perivascular spaces, deep intracerebral hemorrhages and intracerebral calcifications are observed. In familial porencephaly, types 1 and 2 porencephalic cavities following intracerebral hemorrhages in the fetal or neonatal period, periventricular leukoencephalopathy, lacunar strokes and calcifications in neuroimaging are observed. Additional forms of *COL4A1* mutations include infantile hemiparesis, epileptic seizures, migraine with aura, single or recurrent intracerebral hemorrhages, ocular symptoms (tortuous retinal vessels, congenital cataract, an anomaly of the anterior segment of the eye of the Axenfeld-Rieger type), less often with muscle spasms [76].

### 3.12. Cerebral Cavernous Malformations

Cerebral cavernous malformations (also known as cavernous angiomas or cavernomas) are the second most common vascular malformation, with a 0.8% prevalence in the general population [78]. CCMs are a collection of abnormal multiple mulberry-like distended caverns of dilated thin-walled capillaries without the normal intervening brain parenchymal architecture. Often, individual cavernomas are surrounded by hemosiderin, representing the bleeding in the past. The cerebral cavernous malformations can be familiar or sporadic. The familiar form is autosomal dominant with incomplete penetration associated with mutations in KRIT1 (Krev/Rap1 Interacting Trapped 1), *CCM2/MGC4607*, and *CCM3/PDCD10* genes. In most cases CCMs are symptomatic. The clinical picture includes seizures (40–70%), focal neurological symptoms without bleeding (25–50%) and headache (10–30%) and in 25–32% intracranial hemorrhage [79,80,81].

### 3.13. Hereditary Cerebral Haemorrhage with Amyloidosis (HCHWA)

CAA (cerebral amyloid angiopathy) is responsible for 15% of hemorrhagic strokes, and the hereditary form is rare. The genetic form of cerebral amyloid amyloidosis (CAA) is connected with mutations in the gene coding for the amyloid precursor protein. The mutations in genes *CST3*, *BRI*, *TTR*, *ITM2B*, *GSN*, *PRNP* and *ITM2B* are observed [82]. The deposition β-amyloid is connected with destruction of the vessel walls. The progressive degenerative process is due to the development of microaneurysms, as well as hemorrhagic and ischemic lesion of the brain. The specific mutations causes the Dutch, Icelandic or Iowa type of CCA. HCHWA-D is a Dutch type of hereditary cerebral hemorrhage with amyloidosis, which is autosomal dominant condition, caused by a mutation in the amyloid-β precursor gene (Glu693Gl mutation in AβPP) on chromosome 21 leading to amyloid accumulation in the brain. The clinical picture includes recurrent lobar and subarachnoid hemorrhage and lobar micro-hemorrhages before 50 years of age [83]. The Icelandic type of hereditary cerebral hemorrhage with amyloidosis (HCHWA-I) is an autosomal-dominant condition secondary to a mutation in the cystatin gene, which is located on chromosome 20 [84]. The Icelandic type usually occurs earlier and causes intracranial hemorrhage between the ages of 20 and 30.

## 4. Conclusions

Monogenic diseases are rare, but play a significant role in the causes of strokes, especially in young people. The genetic condition connected with strokes could be based on the mutation of a single gene or a cluster of genes with an autosomal-dominant recessive pattern. The clinical features of genetic conditions associated with strokes include a broad spectrum of symptoms. The diagnosis is often very difficult, due to overlapping phenotypes between disorders and the heterogeneity of phenotypes within families. The monogenic causes of stroke are recognizable by key clinical features and radiographic pictures. Genetic tests are expensive but should be part of a routine diagnostic procedure in younger patients with cerebrovascular events, especially in the absence of typical vascular risk factors.

## Figures and Tables

**Table 1:** Single-gene disorders associated with strokes.

	Model of Inheritance	Gene	Stroke Mechanism
CADASIL	AD	*NOTCH-3*	small-vessel disease
CARASIL	AR	*HTRA-1*	small-vessel disease
RVCL	AD	*TREX1*	small-vessel disease
MELAS	Maternal	mitochondrial DNA	multi-factoral
Ehlers-Danlos syndrome type IV	AD	*COL3A1*	arterial dissection
Homocystinuria	AR	*CBS*	small-vessel disease
Fabry disease	X-linked	*GLA*	small-vessel disease
Pseudoxanthoma elasticum (PXE)	AR	*ABCC6*	large-artery disease/ small-vessel disease,
Marfan syndrome	AD	*FBN1*	cardioembolism and arterial dissection
Sickle-cell disease	AR	*HBB*	large-artery disease/small-vessel disease/haemodynamic mechanism
CAA	AD	*APP, CST3*	haemorrhagic stroke
HCHWA-D	AD	*APP*	haemorrhagic stroke
COL4A1 syndrome	AD	*COL4A1*	haemorrhagic stroke
Cerebral cavernous malformations	AD	*CCM1/KRIT1, CCM2/MGC4607, CCM3/PDCD10*	haemorrhagic stroke

AD—autosomal dominant; AR– autosomal recessive; CADASIL—cerebral autosomal dominant arteriopathy with sub-cortical infarcts and leukoencephalopathy; CARASIL—cerebral autosomal recessive arteriopathy with sub-cortical infarcts and leukoencephalopathy; RVCL—retinal vasculopathy and cerebral leukodystrophy; MELAS—mitochondrial myopathy, encephalopathy, lactacidosis, and stroke; mtDNA—mitochondrial DNA; CAA—cerebral amyloid angiopathy; HCHWA-hereditary cerebral hemorrhage with amyloidosis; APP—amyloid-β precursor gene.
